# Clinical Factors Affecting Survival in Patients with Congenitally Corrected Transposition of the Great Arteries: A Systematic Review and Meta-Analysis

**DOI:** 10.3390/jcm13113127

**Published:** 2024-05-27

**Authors:** Sonia Alicja Nartowicz, Ewelina Jakielska, Piotr Ratajczak, Maciej Lesiak, Olga Trojnarska

**Affiliations:** 1 1st Department of Cardiology, Poznan University of Medical Sciences, 61-848 Poznan, Poland; ewelinajakielska@gmail.com (E.J.); maciej.lesiak@skpp.edu.pl (M.L.); olgatroj@wp.pl (O.T.); 2 Department of Pharmacoeconomics and Social Pharmacy, Poznan University of Medical Sciences, 61-701 Poznan, Poland; p_ratajczak@ump.edu.pl

**Keywords:** congenitally corrected transposition of the great arteries, cc-TGA, long-term survival

## Abstract

**Background:** Congenitally corrected transposition of the great arteries (cc-TGA) is a defect characterized by arterio-ventricular and atrioventricular disconcordance. Most patients have co-existing cardiac abnormalities that warrant further treatment. Some patients do not require surgical intervention, but most undergo physiological repair or anatomical surgery, which enables them to reach adulthood. **Aims:** We aimed to evaluate mortality risk factors in patients with cc-TGA. **Results:** We searched the PubMed database and included 10 retrospective cohort studies with at least a 5-year follow-up time with an end-point of cardiovascular death a minimum of 30 days after surgery. We enrolled 532 patients, and 83 met the end-point of cardiovascular death or equivalent event. As a risk factor for long-term mortality, we identified New York Heart Association (NYHA) class ≥III/heart failure hospitalization (OR = 10.53; 95% CI, 3.17–34.98) and systemic ventricle dysfunction (SVD; OR = 4.95; 95% CI, 2.55–9.64). We did not show history of supraventricular arrhythmia (OR = 2.78; 95% CI, 0.94–8.24), systemic valve regurgitation ≥moderate (SVR; OR = 4.02; 95% Cl, 0.84–19.18), and pacemaker implantation (OR = 1.48; 95% Cl, 0.12–18.82) to affect the long-term survival. In operated patients only, SVD (OR = 4.69; 95% CI, 2.06–10.71) and SVR (OR = 3.85; 95% CI, 1.5–9.85) showed a statistically significant impact on survival. **Conclusions:** The risk factors for long-term mortality for the entire cc-TGA population are NYHA class ≥III/heart failure hospitalization and systemic ventricle dysfunction. In operated patients, systemic ventricle dysfunction and at least moderate systemic valve regurgitation were found to affect survival.

## 1. Introduction

Congenitally corrected transposition of the great arteries (cc-TGA) is a congenital heart defect characterized by arterio-ventricular and atrioventricular disconcordance [1]. Systemic venous blood flows into the morphological right atrium, which is connected via the mitral valve to the morphologic left ventricle (LV), the outflow of which is the pulmonary trunk. Therefore, pulmonary venous blood flows into the left morphological atrium, which is connected via the tricuspid valve to the morphologic right ventricle (RV), the outflow of which is the aorta, which makes the right ventricle the systemic pump. The incidence of this complex anatomical defect is estimated at 1 in 33,000 live births, which is approximately 0.05% of all congenital heart defects [2]. The coexistence of additional defects is frequent and present in as many as 90% of patients, the most common of which is ventricular septal defect (VSD, 70%), pulmonary valve stenosis (PS; 40%), and tricuspid valve abnormalities [3,4]. The treatment of choice for cc-TGA depends mainly on the presence of the associated defects. Patients with isolated cc-TGA might not require surgical intervention, and complications resulting from the systemic position of the right ventricle operating under high pressure rarely occur before adulthood [4]. Surgical strategies include a physiological correction in which the right ventricle is maintained as a systemic pump while only the associated lesion is operated. Another approach is anatomic repair utilizing the morphologic left ventricle as a systemic chamber [3]. An example of such an approach, in patients with cc-TGA, hemodynamically significant VSD and PS, or left ventricle outflow tract obstruction (LVOTO), is the Rastelli procedure, during which a patch on VSD is sewn, directing blood from the LV to the aorta, while the RV is connected to the pulmonary artery by a valved conduit [4]. Another anatomical approach is double-switch repair, performed especially in patients with significant tricuspid regurgitation, Ebstein’s tricuspid anomaly, or right ventricular dysfunction [5]. This method changes the direction of venous inflow to the atria with a simultaneous arterial switch, which restores arterio-ventricular and atrioventricular concordance [5]. When biventricular repair is impossible due to unbalanced ventricles, cardiac malposition, and other anatomical lesions, Fontan palliation is performed [6]. Due to these anatomical and surgical consequences, many patients develop common complications like systemic ventricle dysfunction or failure, progressive systemic valve regurgitation, supraventricular and ventricular tachyarrhythmias, and complete atrioventricular block. In addition, less frequent complications may be the consequences of surgical interventions, such as stenosis or regurgitation of the valvular conduit between the RV and the pulmonary artery after Rastelli surgery or baffle stenosis and leakage after anatomic repair. Complications occurring in patients with cc-TGA, described as risk factors for long-term mortality, are supraventricular tachyarrhythmias (SVT), systemic ventricular dysfunction (SVD), systemic valve regurgitation (SVR), and New York Heart Association (NYHA) class ≥ II or complete atrioventricular block. The literature to date consists mainly of single-center studies with a small number of patients, focusing on one of the mentioned surgical approaches. There is a lack of long-term survival analyses taking into account patients who were not operated on and those after physiological or anatomical repair. This meta-analysis is the first study to date assessing risk factors for long-term mortality in patients with cc-TGA.

## 2. Aims

The aim of the study was to evaluate potential long-term mortality risk factors in the group of patients with cc-TGA who were unoperated or were after physiological repair and after anatomical correction.

## 3. Method

### 3.1. Eligibility Criteria

This study used ten retrospective cohort studies of patients with cc-TGA with at least five years (mean/median) follow-up time, with an end-point of long-term mortality. It was defined as follows:

1. Sudden cardiac death (SCD) or equivalent events such as aborted cardiac arrest or appropriate ICD discharge;
2. Non-SCD;
3. Heart transplantation (HTX) at least 30 days after surgery (if applicable).

Selected studies needed to describe the exact reason for mortality and the incidence of the potential risk factors: SVR, SVD, SVT, NYHA class, and the number of implanted pacemakers. We included patients with simple and complex cc-TGA, which was defined as the coexistence of an additional heart lesion: VSD, ASD, PS or atresia, LVOTO, aortic coarctation, Ebstein malformation of the tricuspid valve, or double-outlet right ventricle. All included reports described at least one of the potential risk factors, which were defined as follows:

1. SVT, including regular atrial tachycardia, as well as atrial fibrillation;
2. SVD assessed by an experienced echocardiographer and defined as right ventricular ejection fraction <35% using a visual estimate;
3. Functional NYHA class ≥III or hospitalization due to heart failure (HF);
4. Pacemaker implantation due to atrioventricular block, sick sinus syndrome, or electrical impulses generation disorder;
5. At least moderate SVR, assessed in Doppler echocardiography by an experienced cardiologist.

Protocols with no statistical estimations of association between risk factors and mortality were excluded. We excluded studies focusing on specific subgroups of patients with cc-TGA, e.g., pregnant women or patients only after tricuspid valve replacement. Moreover, additional reasons for exclusion were as follows:

- Studies with less than five patients;
- The language of the manuscript is not English;
- Different articles regarding the previously described group of patients.

Moreover, patients after Fontan palliation were not included in the statistical calculations.

### 3.2. Search Strategy and Study Process

**Figure 1:**
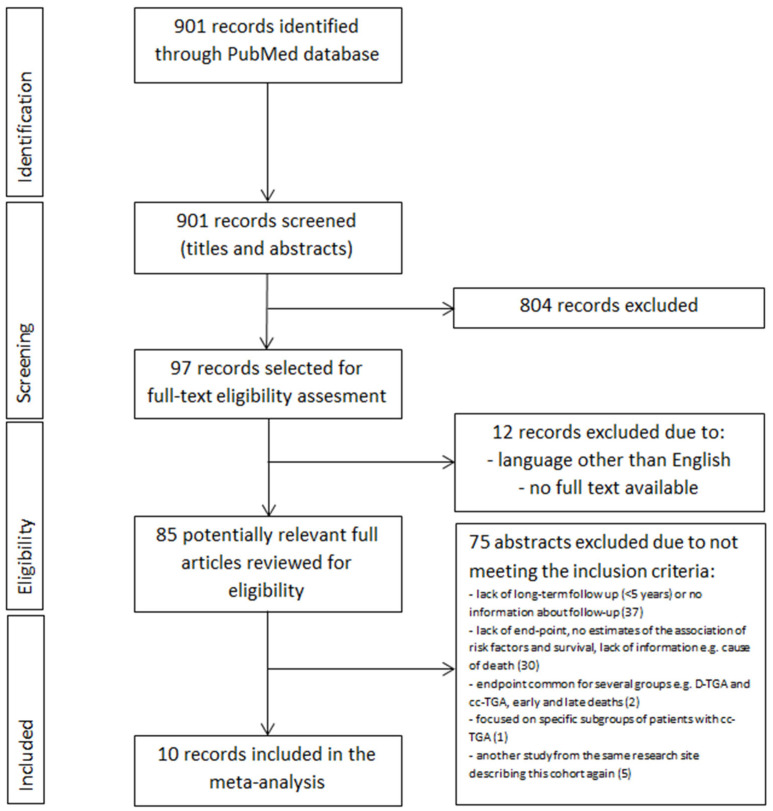
PRISMA summarizing selection process for inclusion of studies. Abbreviations: cc-TGA—congenitally corrected transposition of the great arteries.

The PubMed database was investigated from its inception to 30 November 2022 to find studies of cc-TGA patients describing long-term mortality risk factors. While searching for relevant abstracts, we used the following phrase set: (congenitally corrected transposition of the great arteries OR cc-TGA OR systematic right ventricle) AND (death OR sudden death OR cardiac death OR sudden cardiac death OR outcome OR prognosis OR risk factors) AND (adults OR adolescents)). We did not establish any language restrictions or filters. The first investigator (SN) analyzed the entire database for relevant abstracts while excluding duplicates, from which 97 were selected for full-text eligibility assessment (Figure 1). After excluding 12 analyses due to no full text available and language other than English, investigators one and two (SN and EJ) reviewed 85 potentially relevant complete texts. Eventually, 75 records were excluded for the reasons listed in Figure 1. Therefore, ten studies met the inclusion criteria. Moreover, we reviewed the bibliographies of the included records in search of further potential research. All the studies involved in this analysis were approved by an appropriate ethics committee or institutional review board, and the patient consented to participate. We double-checked all the information included in our study by two investigators. Other team members (PR, ML, and OT) were responsible for substantive care, resolving debatable decisions, and reviewing the methodological correctness of this work. 

### 3.3. Data Extraction and Quality Assessment

Two reviewers (EJ and SN) were responsible for the quality assessment of the included records with the use of the Quality Assessment Tool for Observational Cohort and Cross-Sectional Studies (Table 1) [7]. During the evaluation, we considered whether the number and reason for excluding patients from the analysis and the loss of patients from the study were delivered. We extracted data describing study characteristics such as country, study design, length of follow-up, number of patients in each research group, and number of study sites. The patient population assignment included the percentage of females and males, age, mean or median follow-up time, number of patients with sinus node dysfunction needing pacemaker implantation and reintervention, and the number of subjects after surgery repair and surgery type. We also considered the number of events regarded as long-term mortality: non-SCD, SCD, aborted cardiac arrests, appropriate ICDs, and HTX. 

### 3.4. Statistical Analysis

The included research data were meta-analyzed using Review Manager 5.4. We used the χ2 test to evaluate the significance of heterogeneity between the results of different research and presented it as the I^2^ test. Significant heterogeneity was defined as I^2^ > 50 [8]. The Mantel–Haenszel method and fixed-effect model were used to estimate the odds ratio (OR) of the combined end-point of n-SCD, SCD or SCD equivalent events, and HTX in patients with and without surgical repair in analyses where the heterogeneity turned out to be low and amounted to <50%. The inverse variance method and random effect model were applied in the analysis characterized by high heterogeneity to assess the safety profile of long-term mortality in the described population. The *p*-value < 0.05 was considered statistically significant. We planned to perform a separate meta-analysis for n-SCD and SCD and equivalent events risk factors. However, this was not possible due to the small number of SCDs (n = 17).

A total of 10 retrospective cohort studies were included in this meta-analysis. There were five main comparisons:

1. NYHA functional class ≤ II vs. NYHA ≥ III/HF hospitalization;
2. SVD vs. no SVD;
3. History of SVT vs. no history of SVT;
4. SVR <moderate vs. ≥moderate;
5. Pacemaker vs. no pacemaker implantation.

The secondary end-point was defined as n-SCD, SCD or SCD equivalent events, and HTX only in patients after surgical repair, included five retrospective cohort studies, and consisted of two main comparisons:

1. SVR < moderate vs. ≥moderate;
2. SVD vs. no SVD.

We also planned to perform separate analyses for patients who were not operated on and those operated on with particular surgical methods, but due to the insufficient number of patients and events, it was impossible.

## 4. Results

### 4.1. Study Characteristics

All included trials analyzed a total of 574 subjects. A total of 8 patients were lost to follow-up, and 11 died within less than 30 days after the atrial switch repair. Eighteen patients were excluded from the analysis as they were operated on using the Fontan method. A total of 532 patients were finally enrolled, of which 83 met the combined end-point of n-SCD, SCD or SCD equivalent events, and HTX. All included trials were single-center types from seven countries [9,10,11,12,13,14,15,16,17,18]. The average age in the described population ranged from 9.8 to 42 years, and men accounted for 36.4–67.5%, with an average follow-up time of 5.3– 20 years. The mean age at the time of surgical repair ranged from 2.6 to 13.8 years. A total of 238 patients were operated on in one study; the number of patients after surgery was not reported [14]. Out of patients who had undergone any surgical repair, 64 had a physiological repair, 23 had the Rastelli procedure, and 40 had a double-switch repair. Additionally, 36 patients had a palliative procedure, including Blalock–Taussig shunt, Pott’s and Glenn’s anastomosis, pulmonary artery banding, and the Blalock–Hanlon method. In the rest of the cases, the surgery type was not provided [10,14]. Two studies included in the analysis described populations that had not undergone any surgery [16,17]. Of these 83 deaths, 57 (68.7%) were n-SCD, 16 (19.3%) were SCD, and 10 (12.0%) were HTX. There were 14 SCD equivalent events; however, they occurred in patients who finally died and met the combined end-point. We summarized the characteristics of the included studies in Table 1 and Table 2.

### 4.2. Meta-Analysis

**Figure 2:**
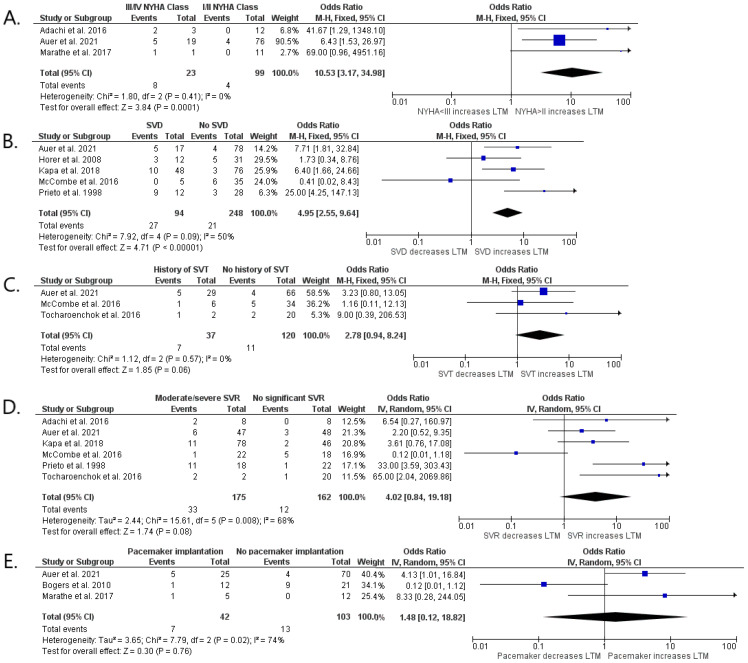
Forest plots showing pooled odds ratios of NYHA >II/heart hospitalization (**A**), at least moderate SVR (**B**), SVD (**C**), history of SVT (**D**), and pacemaker implantation (**E**) for long-term mortality using a random effects meta-analysis approach. Abbreviations: cc-TGA—congenitally corrected transposition of the great arteries; NYHA—New York Heart Association; SVR—systemic valve regurgitation; SVD—systemic ventricular dysfunction; SVT—supraventricular tachyarrhythmia [9,10,12,13,14,15,16,17,18].

**Figure 3:**
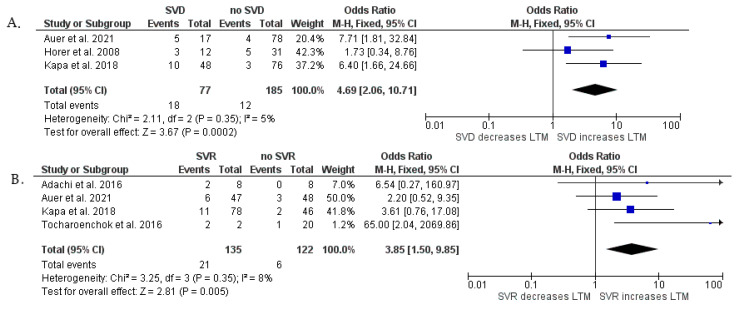
Forest plots showing pooled odds ratios of SVD (**A**) and history of SVR (**B**) for long-term mortality after surgical repair using a random effects meta-analysis approach. Abbreviations—see Figure 2A [9,10,13,14,18].

We performed a meta-analysis of the reported risk factors (Figure 2 and Figure 3). Data on NYHA class ≥III/HF hospitalization were achievable in three publications with a total number of 122 patients [9,10,15], SVT in three types of research with a total number of 157 patients [10,16,18], at least moderate SVR in six articles with 337 patients [9,10,14,17,18], SVD in five studies with 342 patients [10,13,14,16,17], and implantation of pacemaker in three trials with 145 subjects [10,12,15] (Table S1).

At least moderate NYHA class ≥III HF hospitalization (*p* < 0.001) and SVD (*p* < 0.001) were found to have a significant relationship with the combined end-point of n-SCD, SCD, and HTX. We showed a statistical tendency between SVT (*p* = 0.06) and the primary end-point. No relationship was observed between pacemaker implantation (*p* = 0.76), as well as SVR (*p* = 0.08), and the primary end-point (Figure 2). An additional analysis was provided in which we assessed SVD and SVR separately as risk factors of non-SCD, SCD, and HTX, including patients only after surgical repair (Figure 3). This meta-analysis showed that SVD is strongly associated with long-term mortality (OR = 4.69; 95% Cl, 2.06–10.71; *p* < 0.001), as well as SVR (OR = 3.85; 95% Cl, 1.50–9.85; *p* = 0.005). We found that the overall heterogenicity was significant for SVR (I^2^ = 68%) and pacemaker implantation (I^2^ = 74%). The heterogeneity was non-significant for NYHA class ≥ III/HF hospitalization (I^2^ = 0%) and history of SVT (I^2^ = 0%). At least moderate SVD analysis showed intermediate heterogeneity (I^2^ = 50%). Analysis in patients after surgical repair showed low heterogenicity for SVD (I^2^ = 5%) and SVR (I^2^ = 8%).

### 4.3. Sensitivity Analysis

Sensitivity analysis was performed due to high heterogeneity for SVR (I^2^ = 68%) and pacemaker implantation (I^2^ = 74%) in operated and unoperated patients (Figure 2). To obtain the highest quality of results, we also performed a sensitivity analysis for SVD (I^2^ = 50%) in all patients (Figure 2) due to the border value of heterogeneity. The initial analysis of the impact of SVR and SVD on long-term mortality considering both operated and non-operated patients in one group was modified by creating two subgroups. The first group (I) includes patients after surgical correction only, while the second group (II) includes patients not operated on by any method (Figures S1 and S2). After this analysis, the heterogeneity ratio decreased for subgroup I for both SVR (Inverse Variance OR 3.97 [1.41, 11.13] *p* = 0.009 (95% CI), I^2^ = 8%) [9,10,14,18] and SVD (Fixed Model OR 4.69 [2.06, 10.71] *p* < 0.001 (95% CI), I^2^ = 5%) [10,13,14]. High heterogeneity was observed in subgroup II for SVR (Inverse Variance OR 2.03 [0.01, 483.63] *p* = 0.8 (95% CI), I^2^ = 92%), and SVD (Fixed Model OR 5.55 [1.81, 16.99] *p* = 0.003 (95% CI), I^2^ = 82%) [16,17]. Moreover, we performed an additional analysis concerning the influence of pacemaker implantation on long-term mortality in operated patients only (Figure S3). The first subgroup included patients operated by any surgical method [10,12,15], while the second subgroup contained operated patients excluding those after the Rasteli procedure [10,15]. After this analysis, the heterogeneity ratio for the long-term mortality in the II subgroup was reduced (Inverse Variance OR 4.58 [1.25, 16.77] *p* = 0.71 (95% CI), I^2^ = 0%).

## 5. Discussion

Our meta-analysis proved that the function of the systemic chamber and NYHA functional class impact long-term survival in patients with cc-TGA. Systemic valvular regurgitation, supraventricular arrhythmias, and pacemaker implantation do not determine survival in this population. In patients treated surgically, the only two factors affecting survival were systemic ventricle dysfunction and the associated valve regurgitation.

### 5.1. NYHA Class

Our analysis shows that the NYHA > II functional class is a significant risk factor for long-term mortality in patients with cc-TGA. The importance of such a degree of subjective assessment of cardiac function has been confirmed so far in three extensive studies [10,19,20]. Importantly, similarly to the majority of patients with congenital heart anomalies who are never fully healthy, about 80% of these patients, both operated and not operated, assess their physical capacity as well as NYHA I or II. It is, therefore, understandable that a functional state defined by them as greater than NYHA II is associated with an 18-fold increase in the risk of death [10]. Although in the group of patients with cc-TGA, Breda et al. proved a significant correlation between the spiroergometrically assessed objective assessment of physical capacity and the subjective NYHA assessment determined by them, in the conducted study, the majority of subjects did not reach the desired peak VO2 level, which makes the conclusions less credible [21]. Most authors, however, do not confirm such a relationship [4,5,6]. Fredriksen et al. proved that the analyzed patients who obtained only 30–50% of the results achieved by healthy people in the spiro ergometer test describe their physical capacity as NYHA I or II [22]. Therefore, the negative prognostic significance of the deterioration of subjective functional capacity above NYHA II presented in our study should be a particular indication for intensifying the care of these patients. Their objective clinical condition is significantly worse.

### 5.2. Systemic Ventricle Dysfunction

One of the most common complications and causes of death in patients with cc-TGA, accounting for 30–43%, is dysfunction of the systemic ventricle, regardless of the methods of treatment, which was also confirmed by our meta-analysis [10,14,17]. Evidence for this thesis was provided by Prieto et al. after 20 years of observation of 40 young patients and Kapa et al. after over seven years of follow-up of a very large group of 129 patients [14,17]. A similar conclusion by Auer et al. was based on analyzing a less homogeneous group, of which 48% were patients after physiological surgery [10]. In all the mentioned studies, the researchers included only patients whose systemic pump was the right ventricle. The authors agree that the dysfunction of the anatomical right ventricle in patients with cc-TGA results from the load from overcoming the systemic pressure to which it is not adapted [23,24]. Its wall comprises three spatially related, thin layers of muscle that enable peristaltic movement, sufficient to move blood to the low-pressure pulmonary circulation [25]. The need to pump into the systemic circulation causes only a slight thickening of the myocardium. At the same time, a significant dilation of the ventricle leads to functional tricuspid regurgitation, causing its additional volumetric load, which will be discussed later in the paper [26]. The anatomical dysfunction of the right ventricle is additionally aggravated by inadequate coronary blood supply [27]. Patients with complex forms of cc-TGA undergoing correction of additional anatomical anomalies have a worse prognosis due to complications and postoperative residuals [28]. Myocardial scars are the source of ventricular arrhythmia and are particularly important [28]. A separate group of patients is those who underwent a double-switch (anatomical) operation in childhood, in which, after preconditioning, the anatomically left ventricle occupies the systemic position. The study by Anzai et al. shows that it is also subject to inevitable dysfunction [29]. In patients operated in this way, dangerous ventricular arrhythmias are also observed in the short period after the procedure [30]. Tocharoenchok et al. observed the population undergoing this operation, showing that only 64.2% of the subjects survived 10 years after the operation [18]. As a result, the double-switch procedure is performed only in rare, selected cases in children.

### 5.3. Systemic Valve Regurgitation

Our meta-analysis showed that tricuspid regurgitation is a factor of poor prognosis only in operated patients but not in the entire population of patients with cc-TGA. This conclusion is consistent with the observations of Kapa et al. mentioned earlier in this paper [14]. The importance of tricuspid regurgitation in the discussed population is evidenced by the high (56–66%) frequency of its occurrence to a greater than a moderate degree, as well as the frequent coincidence of this phenomenon with the patient’s death [9,16,18]. Tricuspid regurgitation in cc-TGA usually has a complex cause. It may result from a congenital anomaly of the valve leaflets, sometimes with their displacement resembling Ebstein’s syndrome, and a change in the geometry of the complex tricuspid valve apparatus resulting from the dilatation of the right systemic ventricle described above. Long-term significant regurgitation gradually increases the volume load of both right heart chambers, inevitably leading to increased dysfunction and progressive failure of the right ventricle [26,31,32]. The lack of prognostic significance of tricuspid regurgitation in the context of the importance of right ventricular dysfunction can probably be explained by the fact that this damage to the systemic ventricle has not yet occurred. Such an observation may confirm the earlier consideration of surgical correction of this valve when the right ventricle is still functional [4]. On the other hand, the meta-analysis shows a significant prognostic significance of tricuspid regurgitation in patients undergoing surgery. The reason for this dependence is probably their worse hemodynamic condition at the beginning, as well as complications and postoperative remnants, mainly in the form of myocardial scars [6,33]. Therefore, it is understandable that most authors argue that tricuspid regurgitation has a negative prognostic value in all patients with this defect, regardless of the fact of surgery [10,17,34].

### 5.4. Supraventricular Arrhythmia

Even though supraventricular arrhythmia is a frequent and dangerous complication in adult patients with congenital heart defects, especially those with systemic right ventricles, our meta-analysis showed no association with long-term mortality in patients with cc-TGA [4,35,36]. Among the few studies available in the literature dealing with this relationship, our results are confirmed by Dobson et al. based on a 40-year observation of adults (aged 23 to 46) of 32 patients with cc-TGA, in whom supraventricular arrhythmia was diagnosed in as many as 81.3% of the group [37]. On the other hand, Koyak et al. are of the opposite opinion, proving that the presence of a systemic right ventricle is a significant risk factor for death. In the analyzed group, however, only 19% were patients with cc-TGA, which reduces the value of the study’s thesis for this group of patients [38]. These conclusions and the generalizations resulting from the study are also hindered by the heterogeneity of the supraventricular arrhythmia occurring in these patients. In a study conducted by Tseng et al., among 40 patients with cc-TGA, as many as 27 had supraventricular arrhythmias. The most common were atrial flutter (44%), atrial fibrillation (30%), ectopic atrial tachycardia (22%), and, less frequently, multifocal atrial tachycardia (4%) [35]. The cause of this arrhythmia is also complex. These are the extensive scars of the atrial myocardium left after cardiac surgery, which creates conditions for re-entry circulation [35], as well as electrical inhomogeneity of the atrial myocardium stretched as a result of an increase in the end-diastolic pressure of the systemic right ventricle [4,39]. In a non-physiological atrioventricular relationship in patients with cc-TGA, fast, conducted supraventricular rhythm may cause severe hemodynamic disturbances leading to sudden death [16,38]. For this reason, in this unique anatomical anomaly of the heart, frequent, according to Fischbach et al., 20–30% atrioventricular block may be paradoxically beneficial [14,40].

### 5.5. Pacemaker

Although conduction disturbances, including complete atrioventricular block, are a frequent and increasing with age (2% per year) phenomenon, affecting as many as 30–38% of patients with cc-TGA, the fact of constant electrical stimulation of the heart turned out to be not significantly related to long-term mortality in this population [41,42]. Therefore, this treatment method is an effective therapy in treating conduction disorders in the analyzed population.

## 6. Study Limitations

The fundamental limitation of the study is the small size of the study groups, which is typical for the adult population with congenital heart defects, the large anatomical diversity of patients with corrected transposition of the great arteries, and the multitude of surgical methods used. The analysis of the data contained in the studies included in the study is also hindered by the lack of a uniform and precise definition for the complex form of cc-TGA, the diverse methodology of assessing the function of the right ventricle in the systemic position and tricuspid regurgitation, and the apparent subjectivity of the NYHA scale. Demographic differences, the presence of concomitant diseases, the length of the observation, and the level and method of their treatment in individual research centers are significant and evident in this rare group of patients. The studies of patients with cc-TGA included in the meta-analysis, like most of the studies on adults with congenital heart disease in adults, are retrospective, hence the different study plans, definitions, and methods of assessing potential risk factors and endpoints. The aforementioned small size of the study population allowed only univariate analysis without considering the coexistence of individual risk factors.

## 7. Conclusions

The function of the systemic chamber and the resulting exercise capacity assessed by the New York Heart Association functional class have the most significant impact on long-term prognosis in patients with cc-TGA. Systemic valvular regurgitation, supraventricular arrhythmias, and pacemaker implantation do not determine survival in this population. In surgical patients, risk factors for death were systemic ventricle dysfunction and the associated valve regurgitation. However, the described results should be considered with caution due to the mentioned limitations of the work.

## Figures and Tables

**Table 1:** Summary of pooled odds ratios results in random effect model. Abbreviations: CI—confidence interval; HF—heart failure.

Comparison	Odds Ratio	95% Cl	*p*-Value
NYHA functional class >II/HF hospitalization vs. NYHA class <III	10.53	3.17–34.98	*p* < 0.001
At least moderate SVR vs. no SVR	4.02	0.84–19.18	*p* = 0.08
SVD vs. no SVD	4.95	2.55–9.64	*p* < 0.001
History of SVT vs. no history of SVT	2.78	0.94–8.24	*p* = 0.06
Pacemaker vs. no pacemaker	1.48	0.12–18.82	*p* = 0.76
SVD vs. no SVD after surgical repair	4.69	2.06–10.71	*p* < 0.001
SVR vs. no SVR after surgical repair	3.85	1.50–9.85	*p* = 0.005

**Table 2:** Baseline characteristics of included studies reporting late mortality data.

Study	Year Published	Age at Last Follow-Up (Mean) (y)	Follow-Up Duration (Mean/Median) (y)	Lost to Follow-Up for	Number of Patients Included in Analysis	Single or Multi-Center	Mean Age of Cohort at Surgery	Time Period of Surgeries	Proportion of Patients with Anatomic Repair (%)	Proportion of Patients with Rasteli Repair (%)	Proportion of Patients with Fontan Repair (%)
Adachi et al [9].	2016	N/A	19.4	0	16	S	6.9	1970–2000	N/A	75%	0
Auer et al [10].	2021	32.8	6.5	0	96	S	N/A	N/A	N/A	N/A	N/A
Bjarke et al [11].	1976	9.8	6.7	N/A	101	S	N/A	1950–1970	N/A	N/A	0
Bogers et al [12].	2010	N/A	12	3	32	S	13.8	1972–2008	N/A	34.7	0
Horer et al [13].	2008	N/A	7.2	3	56	S	8.6 Fontan Repair 13.4 Classic Repair 3.5 Anatomic Repair	1978–2006	10.7	N/A	19.6
Kapa et al [14].	2018	42	7.2	N/A	129	S	N/A	N/A	N/A	N/A	N/A
Marathe et al [15].	2017	N/A	8.7 Anatomic Repair 6.3 Fontan Repair	0	19	S	7.2 Fontan Repair 2.6 Anatomic Repair	1998–2016	63.2	N/A	36.8
McCombe et al [16].	2016	32.3	13.9	N/A	39	S	N/A	N/A	N/A	N/A	N/A
Prieto et al [17].	1998	25	20	N/A	40	S	N/A	1958–1990	N/A	N/A	N/A
Tocharoenchok et al [18].	2016	10.9	5.3	2	22	S	10.9	2001–2014	100	0	0

## Data Availability

No data were provided.

## Supplementary Materials

The following supporting information can be downloaded at https://www.mdpi.com/article/10.3390/jcm13113127/s1, Figure S1: Sensitivity analysis for systemic valve regurgitation. Figure S2: Sensitivity analysis for systemic ventricle dysfunction. Figure S3: Sensitivity analysis for pacemaker implantation. Table S1: Primary and key secondary outcomes of included studies reporting late mortality. Abbreviations: AV block—atrioventricular block; CVD—cardiovascular death; HTX—heart transplantation; other—see Figure 2.
