# Supplementary material for: Clinical Factors Affecting Survival in Patients with Congenitally Corrected Transposition of the Great Arteries: A Systematic Review and Meta-Analysis
Source: J Clin Med. 2024 May 27;13(11):3127. doi: 10.3390/jcm13113127 (PMC11173277; doi:10.3390/jcm13113127)
Supplement: Supplementary file 1 [file jcm-13-03127-s001.zip › jcm-2939814-supplementary.pdf]

Journal of
Clinical Medicine

MDPI

Table S1. Primary and key secondary outcomes of included studies reporting late mortality. Abbreviations: AV block—atrioventricular block; CVD—cardiovascular death; HTX—heart transplantation; other—see Figure 2.

Study	SCD	CVD	HTX	Long-term mortality (or equivalent events in total)	SVT incidence (%)	AV block incidence (%)	Pacemaker implantation incidence (%)	NYHA Class > II incidence (%)	SVD incidence (%)	SVR incidence (%)	Reintervention incidence (%)	Annual long-term mortality (%/year)
Adachi et al.	0	2	0	2	N/A	N/A	N/A	18.8	N/A	50	37.5	0.1
Auer et al.	0	9	0	9	30.2	N/A	26	19.8	17.7	49	N/A	1.4
Bjarke et al.	2	17	0	19	7.9	16.8	0	N/A	24.8	N/A	1	2.8
Bogers et al.	0	7	3	10	N/A	N/A	41.4	21.9	70	62.5	37.5	1.7
Horer et al.	0	6	2	8	N/A	16.1	26.8	N/A	22	4.9	35.7	1.1
Kapa et al.	10	3	0	13	N/A	N/A	N/A	N/A	43.4	60.5	N/A	1.8
Marathe et al.	0	0	1	1	N/A	31.6	36.8	5.3	N/A	N/A	21.2	*0.1 Anatomic Repair 0.2 Fontan Repair*
McCombe et al.	5	1	1	6	15.4	35.9	20.5	N/A	12.8	56	N/A	0.4
Prieto et al.	0	9	3	12	N/A	32.5	N/A	N/A	30	42.5	N/A	0.6
Tocharoenchok et al.	0	3	0	3	9.1	N/A	N/A	9.1	N/A	9	27.3	0.6

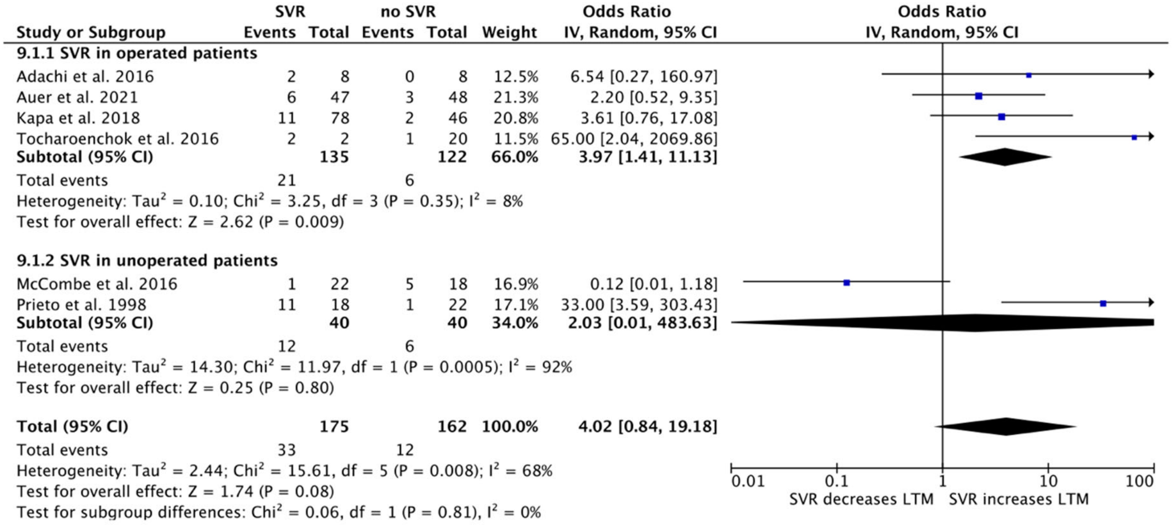

Figure S1. Sensitivity analysis for systemic valve regurgitation.

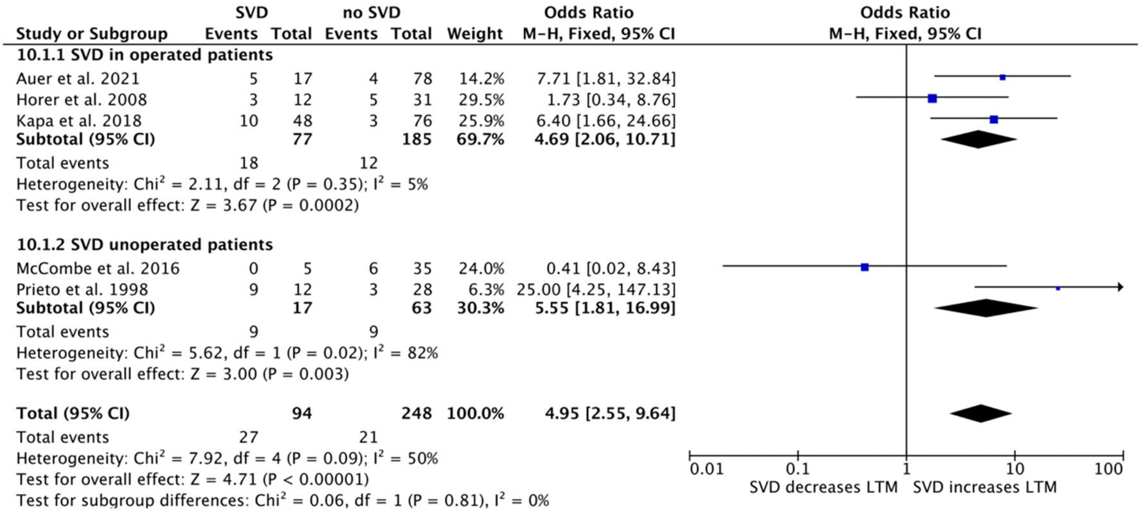

Figure S2. Sensitivity analysis for systemic ventricle dysfunction.

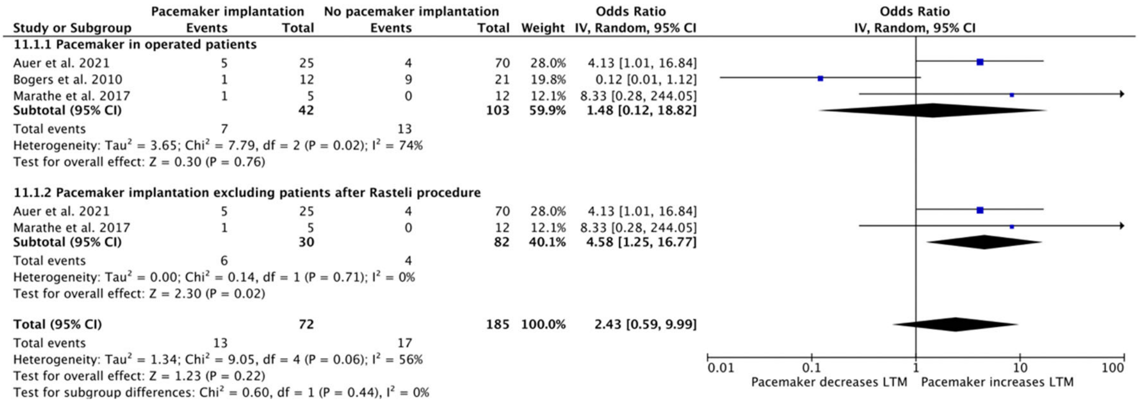

Figure S3. Sensitivity analysis for pacemaker implantation.
